# Room-Temperature Synthesis and Photodetector Performance of All-Inorganic Lead-Free Cs_**3**_Bi_**2**_ *X*_**9**_ (*X* = Cl, Br, I) Perovskite Nanocrystals

**DOI:** 10.1021/acsami.5c11230

**Published:** 2025-10-22

**Authors:** Minal Chopade, Mahesh Kamble, Komal Gadekar, Shruti Shah, Abhijit Landge, Minal Kurane, Priti Vairale, Shashikant P. Patole, Sandesh Jadkar

**Affiliations:** † Department of Physics, 470175 PDEA’s Prof. Ramkrishna More Arts, Commerce, and Science College, Pune 411044, India; ‡ Center for Energy Studies, 29638 Savitribai Phule Pune University, Pune 411007, India; § Department of Physics, 209969 Ferguson College, Pune 411004, India; ∥ Department of Physics, 105955 Khalifa University of Science and Technology, Abu Dhabi 127788, United Arab Emirates; ⊥ Department of Physics, Savitribai Phule Pune University, Pune 411007, India

**Keywords:** all inorganic Cs_3_Bi_2_ *X*_9_ perovskites, antisolvent recrystallization, XRD, raman spectroscopy, XPS, photodetectors

## Abstract

All-inorganic halide perovskites have gained significant interest as environmentally friendly alternatives to Pb-based perovskites for optoelectronic applications. Here, we report the preparation of Pb-free inorganic Cs_3_Bi_2_ *X*_9_ (*X* = Br, I, Cl) perovskite using a simple room-temperature antisolvent recrystallization method on soda-lime glass substrates. We thoroughly analyzed and compared the structural, optical, and morphological properties of these films. X-ray diffraction analysis revealed that the Cs_3_Bi_2_Cl_9_ perovskite has the largest crystallite size (51 nm) compared to the Cs_3_Bi_2_Br_9_ and Cs_3_Bi_2_I_9_ perovskites. The perovskite films demonstrated enhanced crystallinity and exhibited hexagonal and orthorhombic phases with distinct space groups. Raman spectroscopy confirmed the successful formation of the Cs_3_Bi_2_ *X*_9_ phase. Field emission scanning electron microscopy analysis showed that Cs_3_Bi_2_ *X*_9_ exhibited irregular morphologies with noticeable voids across all samples. The surfaces were predominantly covered with uneven, spherical-like granules. The surface morphology displays dense packing with evident porosity and particle sizes ranging from submicrons to a few micrometers. The elemental analysis of the Cs_3_Bi_2_ *X*_9_ perovskite revealed the formation of nearly stoichiometric films. The light absorption edge of Cs_3_Bi_2_ *X*_9_ varied from 1.8 to 3.2 eV, depending on the halide composition. The formation of Cs_3_Bi_2_ *X*_9_ perovskites was confirmed using X-ray photoelectron spectroscopy analysis. Finally, we fabricated photodetectors based on Cs_3_Bi_2_ *X*_9_. Among these, the Cs_3_Bi_2_Cl_9_ perovskite-based photodetector exhibited superior performance, with a detectivity of 29.6 × 10^8^ Jones, photoresponsivity of 1.14 mA/W, a fast rise time of 0.21 s, a decay time of 0.35 ms, and an internal quantum efficiency of 2.45 × 10^–1^ %. These findings demonstrate the potential of Cs_3_Bi_2_Cl_9_ perovskite-based photodetectors as promising candidates for next-generation, lead-free photodetector devices.

## Graphical abstract

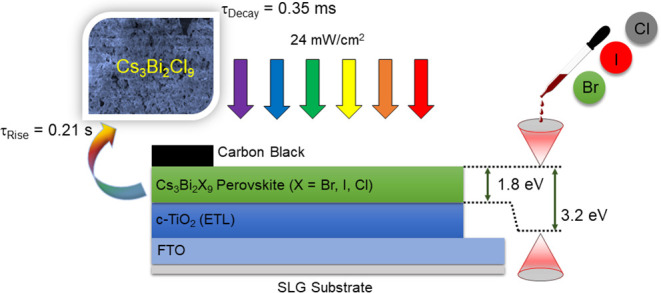

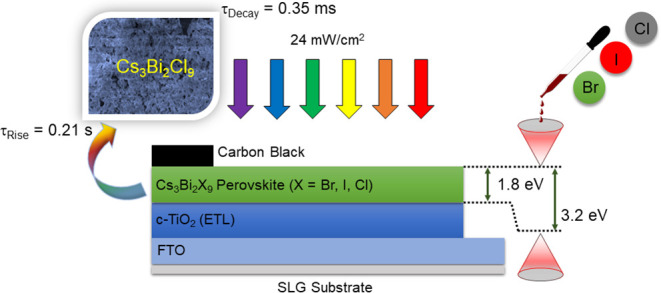

## 1 Introduction

Perovskite materials have garnered significant attention in recent years due to their remarkable optoelectronic properties, making them highly promising for a wide range of applications such as photovoltaics,^1^ LEDs,^2^ photodetectors,^3^ lasers,^4^ and more. Due to several outstanding properties, perovskite compounds containing halide ions have gained significant interest for optoelectronic applications. They exhibit excellent charge carrier mobility, which allows electric charges (electrons and holes) to move quickly through the material, enhancing the device’s performance. Their band gap can be easily tuned by changing the composition, making them suitable for detecting or absorbing light across a wide range of wavelengths. In addition, halide perovskites possess strong light absorption ability, meaning they can absorb significant amounts of light even in very thin layers, which is ideal for trivial and compact devices. The exciton diffusion length describes the range of electron–hole pairs migrating before recombining. This feature enhances charge collection efficiency in devices such as photodetectors and solar cells. Another significant advantage is their simple and low-cost fabrication process. Halide perovskites can be processed using solution-based methods, which are scalable and suitable for large-area device production. These combined properties make halide perovskites promising candidates for high-performance, cost-effective optoelectronic devices.^5–7^

The synthesis of traditional perovskite materials often requires organic solvents, which are harmful to the environment.^8^ It raises concerns about the environmental impact, especially when scaling up production for commercial applications. Furthermore, the most commonly used perovskites are based on lead (Pb), which presents two major drawbacks: toxicity and chemical instability.^9^ These factors limit their practical use in large-scale and long-term optoelectronic devices. Because of these issues, there is a strong need to develop new perovskite materials that are both environmentally friendly and chemically stable without compromising device performance. To address this, researchers have explored replacing toxic Pb with less harmful and more stable elements such as tin (Sn), antimony (Sb), bismuth (Bi), and copper (Cu).^10–13^ Such alternative materials offer a more sustainable and safer option for perovskite-based optoelectronic technologies, including solar cells, photodetectors, and light-emitting devices. Efforts are ongoing to optimize the performance of these Pb-free perovskites so they can meet or even exceed the efficiency and stability of their lead-based counterparts while avoiding the environmental and health risks associated with Pb. Pb-free inorganic halide perovskites, with the general formula A_3_Bi_2_ *X*_9_ (where *X* = Br, I, and Cl), have recently attracted significant attention in the research community mainly due to their excellent chemical stability,^14^ low toxicity, strong photoluminescence, high quantum yield, and tunable band gaps.^15–20^ These properties make them promising candidates as environmentally friendly alternatives to traditional lead-based perovskites. Cs_3_Bi_2_ *X*_9_ (*X* = Br, I, or Cl) has emerged as an attractive material. It combines the benefits of nontoxicity and environmental safety with many desirable characteristics of halide perovskites, such as high light absorption and excellent electronic performance.^14^ Specifically, Cs_3_Bi_2_ *X*_9_ exhibits high absorption coefficients, excellent thermal stability, and a broad spectral response, making it an ideal material for photodetector devices. Due to these advantages, Cs_3_Bi_2_ *X*_9_ has already been explored for various applications, including photodetectors, memory devices, solar cells, and X-ray detectors.^21–26^

Photodetectors are essential in various fields, including medical diagnostics, environmental monitoring, and optical communication systems. To be effective, these devices must quickly and efficiently convert incoming light into electrical signals, which requires materials with high sensitivity, fast response times, and good stability.^27^ While most commercial photodetectors are made using materials like silicon (Si), gallium phosphide (GaP), or lead sulfide (PbS), their fabrication often requires complex and expensive vacuum-based processes, which limit scalability and increase production costs. In contrast, perovskite materials, especially Pb-free Cs_3_Bi_2_ *X*_9_, offer several advantages: high carrier mobility, adjustable band gaps, strong light absorption, and low-cost solution-based synthesis methods^28,29^ that do not require vacuum systems. With favorable optoelectronic properties, eco-friendly composition, and simple processing techniques, Cs_3_Bi_2_ *X*_9_ has excellent potential to improve the performance and affordability of next-generation photodetectors. In the present work, we outline the preparation and analysis of Cs_3_Bi_2_ *X*_9_ (*X* = Br, I, and Cl) perovskites using an antisolvent recrystallization method for photodetector applications. We systematically investigated these halide-based perovskite's structural, morphological and optical properties, focusing on how different halide ions (bromine, iodine, and chlorine) influence their crystallinity, morphology, and light absorption behavior. The performance of the Cs_3_Bi_2_ *X*_9_-based photodetectors is evaluated in terms of key parameters such as photoresponsivity, detectivity, and response time. Our results indicate the promising capability of Pb-free Cs_3_Bi_2_ *X*_9_ perovskite materials in developing high-performance, environmentally friendly photodetectors for next-generation optoelectronic purposes.

## 2 Experimental Details

### 2.1 Materials

Cesium tribromide (CsBr_3_), bismuth tribromide (BiBr_3_), cesium iodide (CsI), bismuth triiodide (BiI_3_), cesium chloride (CsCl), and bismuth trichloride (BiCl_3_) were purchased from Sigma-Aldrich and used directly without further treatment or modification. The solvents used in this study, sourced from HPLC Chemicals, included N, N-Dimethylformamide (DMF, C_3_H_7_NO), Dimethyl Sulfoxide (DMSO, C_2_H_6_OS), Isopropanol (IPA, C_3_H_3_O), Toluene (C_6_H_5_CH_3_), and Acetone [(CH_3_)_2_CO)]. Fluorine-doped tin oxide (FTO) substrates were used for device performance evaluation.

### 2.2 Preparation of Perovskite Precursor and Synthesis of Films

The soda-lime glass (SLG) was used to deposit the Cs_3_Bi_2_ *X*_9_ (*X* = Br, I, Cl) perovskite layers. The schematic illustration of the synthesis process of Cs_3_Bi_2_Br_9_ perovskite using the antisolvent recrystallization method is depicted in Figure 1.

**Figure 1:**
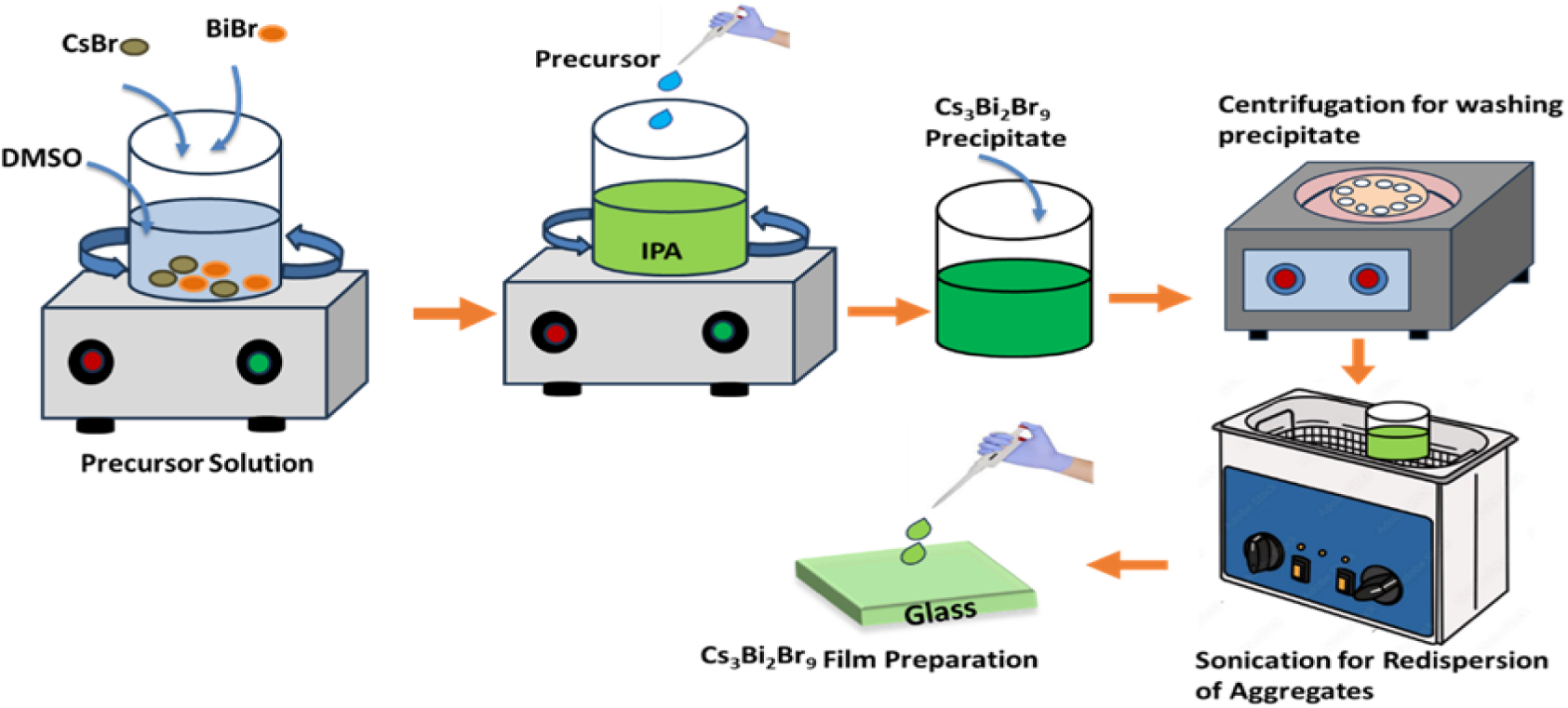
Representation of the synthesis of Cs_3_Bi_2_Br_9_ perovskite using the antisolvent recrystallization method.

To synthesize Pb-free Cs_3_Bi_2_Br_9_ nanocrystals, CsBr_3_ and BiBr_3_ were mixed in a 3:2 molar ratio and dissolved in DMSO to prepare a precursor solution. Specifically, 90 μmol of CsBr_3_ (19.15 mg) and 60 μmol of BiBr_3_ (26.92 mg) were dissolved in 5 mL of DMSO. Then, 400 μL of the precursor mixture was added dropwise to 10 mL of isopropanol, with vigorous agitation for 5 min. The solution turned yellowish-green and was centrifuged at 4500 rpm for 10 min to separate unreacted particles. The resulting clear yellowish-green nanocrystals were washed using a toluene-acetone mixture (8:2 ratio) and centrifuged again. Nanocrystals tend to aggregate during washing due to incomplete removal of excess precursors. Sonication was used to break these aggregates, ensuring a uniform dispersion of nanocrystals in the solvent and producing a smooth, uniform, thin film.

For the synthesis of Cs_3_Bi_2_I_9_ nanocrystals, precursors CsI and BiI_3_ were dissolved in a DMF solvent. The resulting solution was then drop-casted into an antisolvent bath (toluene and acetone) under vigorous stirring to induce recrystallization. The obtained nanocrystals were then washed, sonicated, and deposited on SLG substrates following the same steps as for Cs_3_Bi_2_Br_9_ nanocrystals. A similar procedure was followed for synthesizing Cs_3_Bi_2_Cl_9_ nanocrystals using CsCl and BiCl_3_ precursors with DMSO as solvents and toluene and acetone as antisolvents. All films were dried at room temperature before characterization.

Figure 2 presents the photographs of Cs_3_Bi_2_ *X*_9_ (*X* = Br, I, Cl) perovskite films, and the inset in each shows the corresponding precursor solution.

**Figure 2:**
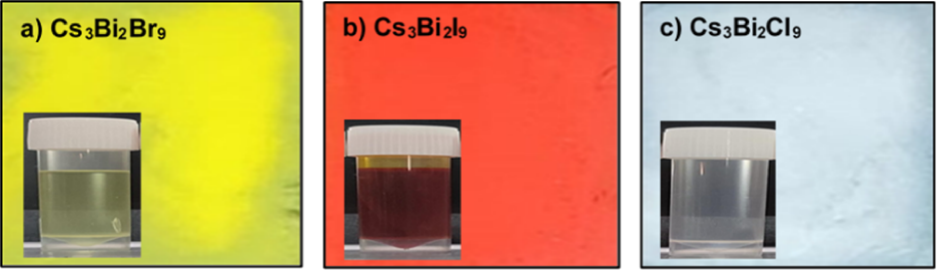
Actual photographs of Cs_3_Bi_2_ *X*_9_ perovskite films (a) Cs_3_Bi_2_Br_9_, (b) Cs_3_Bi_2_I_9_, and (c) Cs_3_Bi_2_Cl_9_. The inset in each photograph shows the corresponding precursor solution.

### 2.3 Material Characterization

The crystal structure of the prepared Cs_3_Bi_2_ *X*_9_ (*X* = Br, I, Cl) films was analyzed with X-ray diffraction (XRD; D8-Advance, Bruker AXS) with a Cu–Kα radiation source (λ = 1.54 Å) at a grazing angle of 1°. Raman spectra were recorded in the 100–300 cm^–1^ range using a Raman spectrometer (Jobin Yvon Horiba LABRAM-HR Raman) with a resolution of 1 cm^–1^ in a backscattering geometry. A He–Ne laser (532 nm) was the excitation source. X-ray photoelectron spectroscopy (XPS) (Thermo Scientific, K Alpha, U.K.) was performed to determine the elemental composition of the films. The surface morphology and chemical composition were examined using field emission scanning electron microscopy (FE-SEM) (Nova Nano SEM 450) equipped with energy-dispersive X-ray spectroscopy (EDAX). The optical band gap of Cs_3_Bi_2_ *X*_9_ was estimated by recording the absorbance spectrum in the 300–700 nm range using a JASCO V-670 UV–visible-NIR spectrophotometer. Room temperature photoluminescence (PL) measurements were conducted using a Fluorolog Horiba Scientific setup.

### 2.4 Preparation of the Electron Transport Layer (ETL)

The compact TiO_2_ (c-TiO_2_) layers were coated onto an FTO-coated glass through the chemical bath deposition (CBD). First, a solution was prepared by mixing 10 mL of titanium trichloride (TiCl_3_) with 40 mL of double-distilled water (DDW) in a beaker. DDW served to dilute a predetermined proportion of TiCl_3_ (15% solution in hydrochloric acid, HCl). The solution was stirred thoroughly to ensure uniform mixing. A 1 M NaOH solution was used to adjust the pH of the solution to between 1 and 2 while ensuring continuous stirring at room temperature. After reaching the desired pH, FTO-coated glass slides were dipped into the solution. The deposition bath temperature was kept constant at 27 °C throughout the reaction. After 72 h, the substrates were removed, rinsed with water to discard any unbound particles, and left to dry under ambient conditions. The films were heat-treated in an oven at 450 °C for 1 h as a final step. The formation of c-TiO_2_ layer on FTO-coated glass substrate using the CBD method was confirmed by low-angle XRD analysis. Figure 3 shows the XRD pattern of the c-TiO_2_ layer deposited on FTO-coated glass substrate.

**Figure 3:**
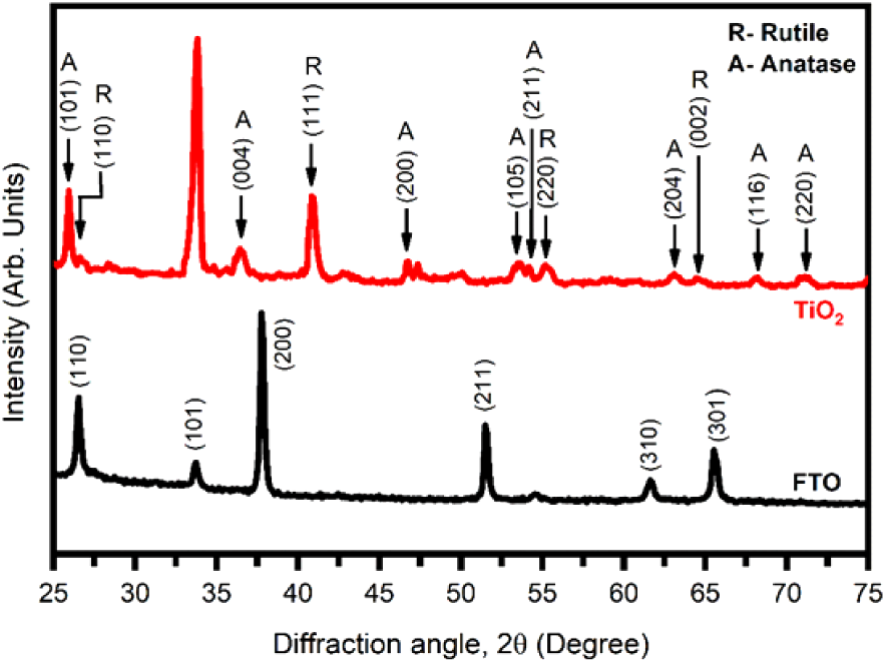
XRD spectra of the c-TiO_2_ layer deposited on FTO-coated glass substrate using the CBD method.

The diffraction peaks at 2θ ∼ 25.3°, 36.4°, 47.4°, 53.9°, 54.1°, 68.4°, 70.8°^,^ and 75.6° corresponding to the (101), (004), (200), (105), (211), (204), (116), and (220) diffraction planes of anatase TiO_2_, respectively, in agreement with the standard JCPDS Data Card #96-900-9087. The diffraction peaks at 2θ ∼ 26.6°, 40.8°, 55.2°, and 68.3° corresponding to the (110), (111), (220), and (002) diffraction planes of rutile TiO_2_, respectively, in agreement with the standard JCPDS Data Card #96-900-9084. The observed XRD peaks at 2θ ∼ 26.4° (111), 33.6° (101), 37.6° (200), 51.4° (211), 61.6° (310), and 65.4° (301) refer to FTO glass.

Hence, the XRD data confirmed the formation of c-TiO_2_ layer on FTO-coated glass substrate using the CBD method.

### 2.5 Device Fabrication

FTO-layered glass substrates were sequentially washed in an ultrasonic cleaner using acetone, isopropanol, anhydrous alcohol, and deionized water for 15 min each. A c-TiO_2_ layer was deposited on the cleaned FTO substrate using the CBD, as described in Section 2.4. The Cs_3_Bi_2_ *X*_9_ perovskite layer was deposited onto the c-TiO_2_ layer using the spin-coating technique at 4000 rpm for 30 s. A carbon black mixture was obtained by adding carbon black powder to isopropanol (IPA). This paste was applied as the top electrode using the doctor blade method. The coated film was dried using a temperature-controlled plate at 60 °C to form the FTO/c-TiO_2_/Cs_3_Bi_2_ *X*_9_/Carbon black photodetector structure. The fabricated photodetector was then used for photoresponse measurements to evaluate its performance.

## 3 Results and Discussion

### 3.1 XRD Analysis

The formation of Cs_3_Bi_2_ *X*_9_ (*X* = Cl, Br, I) perovskite was confirmed using low-angle XRD analysis. The XRD pattern of Cs_3_Bi_2_ *X*_9_ perovskite films deposited on SLG using the antisolvent recrystallization method is shown in Figure 4. Multiple peaks in the XRD pattern confirmed the polycrystalline structure of Cs_3_Bi_2_ *X*_9_ (*X* = Cl, Br, I) nanocrystals. The diffraction peaks matched well with standard JCPDS card #70-0990 for Cs_3_Bi_2_Cl_9_ (Orthorhombic, space group Pmcn), #23-0847 for Cs_3_Bi_2_Br_9_ (Hexagonal, space group P63/mmc), and # 44–0714 for Cs_3_Bi_2_I_9_ (Hexagonal, space group *P*3̅*m*1). The sharp, well-defined diffraction peaks indicated that the synthesized perovskites were highly crystalline.

**Figure 4:**
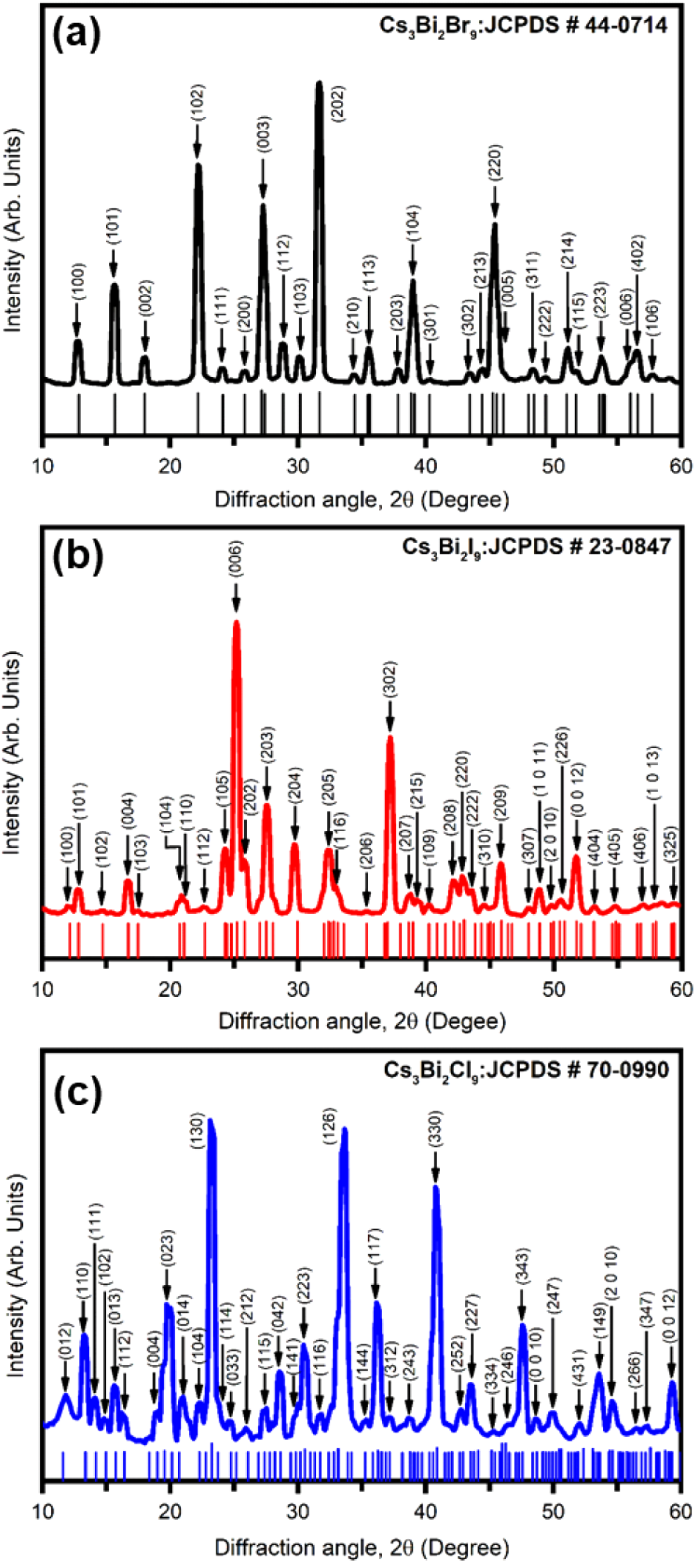
XRD spectra of (a) Cs_3_Bi_2_Br_9_, (b) Cs_3_Bi_2_I_9_, and (c) Cs_3_Bi_2_Cl_9_ perovskite halides along with respective JCPDS data card.

The average crystallite size (*d*_X‑ray_), dislocation density (δ), and microstrain (ε) of Cs_3_Bi_2_ *X*_9_ (*X* = Cl, Br, I) perovskites were calculated using,^30–32^

1 $$d_{x-\mathrm{ray}}=\frac{0.9\times \lambda }{\beta \times \mathrm{Cos}\theta }$$

2 $$\delta =\frac{n}{d_{x‐\mathrm{ray}}^{2}}$$

3 $$\varepsilon =\frac{\beta }{4\times \tan\theta }$$

Where λ is the wavelength of the X-ray source, β is the full width at half-maximum of the diffraction line, and θ is the diffraction angle. The calculated structural parameters of Cs_3_Bi_2_ *X*_9_ perovskites are listed in Table 1.

**Table 1:** Calculated Crystallite Size, Dislocation Density, and Microstrain of Cs_3_Bi_2_ *X*_9_ (*X* = Cl, Br, I) Perovskites

Perovskite	Average crystallite size (nm)	Dislocation density × 10^–4^ (nm^–2^)	Microstrain × 10^–3^ (ε)
**Cs**_**3**_ **Bi**_**2**_ **Br**_**9**_	50	0.40	2.55
**Cs**_**3**_ **Bi**_**2**_ **I**_**9**_	48	0.43	3.26
**Cs**_**3**_ **Bi**_**2**_ **Cl**_**9**_	51	0.38	3.37

All Cs_3_Bi_2_ *X*_9_ nanocrystals exhibited preferred orientation and showed distinct structural characteristics. Cs_3_Bi_2_Cl_9_ showed the highest crystallinity, with sharper peaks and smaller full width at half-maximum (FWHM-), indicating greater crystallite sizes than the bromine- and iodine-based perovskites. In contrast, Cs_3_Bi_2_Br_9_ and Cs_3_Bi_2_I_9_ displayed broader peaks with higher FWHM values, suggesting smaller crystallite sizes. The differences in crystallite size can be attributed to the ionic radii of the halides, where Cl^–^ (the smallest radius) leads to stronger lattice interactions and better crystal packing. In contrast, Br^–^ and I^–^ (larger radii) result in more lattice strain and smaller crystallite sizes. Additionally, the increase in internal strain from iodine to chlorine is due to lattice contraction caused by the decreasing ionic radius. These results align well with previously reported structural data and confirm the successful formation of Cs_3_Bi_2_ *X*_9_ perovskite nanocrystals with tunable structural properties depending on the halide composition.^33–35^

### 3.2 Raman Spectroscopy Analysis

The crystallinity and phase formation of Cs_3_Bi_2_ *X*_9_ perovskite material were further confirmed by Raman spectroscopy. Figure 5 illustrates the Raman spectra of Cs_3_Bi_2_Br_9_, Cs_3_Bi_2_I_9_, and Cs_3_Bi_2_Cl_9_ excited by a 532 nm laser from 100 to 300 cm^–1^.

**Figure 5:**
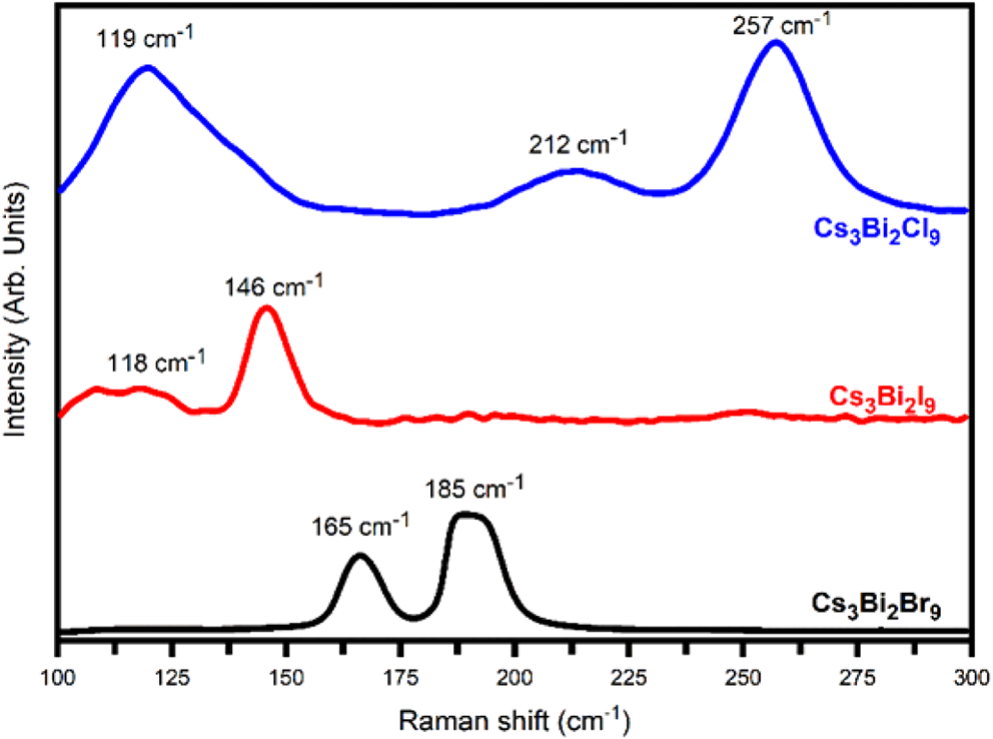
Raman spectra of Cs_3_Bi_2_ *X*_9_ (*X*= Cl, Br, I) perovskites deposited on the glass using antisolvent recrystallization.

The Raman spectral analysis of Cs_3_Bi_2_Br_9_ displays two prominent peaks at ∼165 cm^–1^ and ∼185 cm^–1^. These bands are arising due to the oscillations of Bi–Br bonds in [BiBr_6_] octahedral.^36^ Similarly, a major peak is observed at ∼146 cm^–1^ in the Raman spectrum of Cs_3_Bi_2_I_9_, which is characteristic of Bi–I stretching inside the [BiI_6_]^3–^ octahedron.^37^ In the case of Cs_3_Bi_2_Cl_9_ perovskite, Raman spectra show three major bands at ∼119 cm^–1^, ∼212 cm^–1^, and ∼257 cm^–1^ are related to the T_2g_ (Breathing), E_9_, and A_1g_ (both stretching) vibration modes of the (BiCl_6_)^3–^ octahedron, respectively. The intensity of the Bi–Cl vibrational band in Cs_3_Bi_2_Cl_9_ perovskite is much higher than that of the Bi–Br in Cs_3_Bi_2_Br_9_ and Bi–I in Cs_3_Bi_2_I_9_ perovskites, suggesting that Cs_3_Bi_2_Cl_9_ perovskite has a more ordered structure. These results confirm the presence of metal halide octahedral units in the synthesized Cs_3_Bi_2_ *X*_9_ perovskite nanocrystals.^38^

### 3.3 XPS Analysis

XPS measurements were utilized to analyze the compositional and elemental properties of the Cs_3_Bi_2_ *X*_9_ (*X* = Cl, Br, I) perovskite. The spectrometer dispersion was calibrated using the C 1s line of adventitious carbon at a binding energy of 284.8 eV. Figure 6a represents a typical XPS-wide scan of Cs_3_Bi_2_ *X*_9_ (*X* = Br, Cl, I) perovskite nanocrystals synthesized using the antisolvent recrystallization method. The wide scan shows the peaks of Cesium (Cs), Bismuth (Bi), Bromine (Br), Chlorine (Cl), Iodine­(I), Oxygen­(O), and Carbon (C).

**Figure 6:**
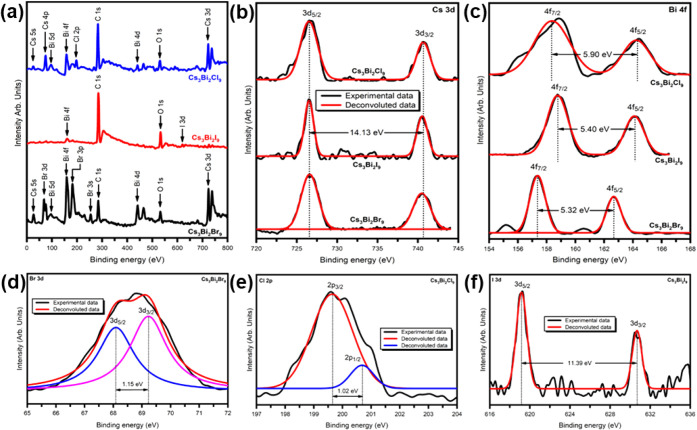
(a) Survey scan XPS spectra for Cs_3_Bi_2_ *X*_9_ (*X* = Br, I, Cl) perovskite nanocrystals. Core Level XPS spectra for (b) Cs 3d, and (c) Bi 4f in Cs_3_Bi_2_ *X*_9_ (*X* = Br, I, Cl) perovskite nanocrystals. Core level spectra for (d) Br 3d in Cs_3_Bi_2_Br_9_, (e) I 3d in Cs_3_Bi_2_I_9_, and (f) Cl 2p in Cs_3_Bi_2_Cl_9_ perovskite nanocrystals.

Figure 6b–f shows the high-resolution XPS spectra of Cs_3_Bi_2_ *X*_9_ (*X* = Br, I, Cl) perovskite nanocrystals. It exhibited peaks signifying Cs 3d, Bi 4f, Br 3d, I 3d, and Cl 2p, confirming the presence of these elements. Figure 6b shows the elemental scan of Cs 3d for Cs_3_Bi_2_Br_9_, Cs_3_Bi_2_I_9_, and Cs_3_Bi_2_Cl_9_, respectively. All halide variants display two spectral lines associated with the spin–orbit split components Cs 3d_5/2_ and Cs 3d_3/2_. The peak located at ∼726.56 eV is from 3d_5/2_ Cs, while another at ∼740.69 eV corresponds to 3d_3/2_ Cs with a spin–orbit splitting of 14.13 eV, indicating that Cs has a + 1 oxidation state.^39^ For Bi, the peaks observed at ∼158.37, 158.78, and 157.36 eV correspond to Bi 4f_7/2_ in Cs_3_Bi_2_Cl_9_, Cs_3_Bi_2_I_9_, and Cs_3_Bi_2_Br_9_, respectively, whereas peaks observed at ∼164.30, 164.15, and 162.68 eV correspond to Bi 4f_5/2_ in Cs_3_Bi_2_Cl_9_, Cs_3_Bi_2_I_9_, and Cs_3_Bi_2_Br_9_, respectively. These results suggest that Bi has a + 3 oxidation state. The deconvoluted XPS spectra of Br 3d show two major peaks at ∼68.05 eV and ∼69.20 eV associated with Br 3d_5/2_ and 3d_3/2_ with an energy separation of 1.15 eV, indicating Br has a −1 oxidation state in Cs_3_Bi_2_Br_9_ perovskite.^15–41^ The I 3d_5/2_ and I 3d_5/2_ binding energy peaks appear at ∼619.0 and 630.39 eV with a spin–orbit splitting of 11.39 eV, characteristic of iodide compounds.^42^ The core level XPS spectrum for Cl shows peaks at ∼199.5 eV for 2p_3/2_ and another at ∼200.6 eV for 2p_1/2_ states with 1.02 eV energy separation. Table 2 presents the core-level binding energy peak positions and atomic percentages of elements identified in the synthesized Cs_3_Bi_2_ *X*_9_ (*X* = Cl, Br, I) perovskite nanocrystals. These values were obtained using XPS analysis, which confirmed the elemental composition of the materials synthesized via the antisolvent recrystallization method.

**Table 2:** XPS Energy Peak Positions of Elements in Cs_3_Bi_2_ *X*_9_ (*X* = Cl, Br, I) Perovskite Nanocrystals Synthesized via the Antisolvent Recrystallization Method

Elements	Core level	Peak position in Cs_3_Bi_2_Br_9_ (eV)	At. %	Peak position in Cs_3_Bi_2_I_9_ (eV)	At. %	Peak position in Cs_3_Bi_2_Cl_9_ (eV)	At. %
Cs	3d_5/2_	∼726.56	10.62	∼726.54	11.55	∼726.54	17.44
	3d_3/2_	∼740.69	14.79	∼740.59	12.90	∼740.69	12.88
Bi	4f_7/2_	∼157.36	15.74	∼158.78	45.60	∼158.37	07.13
	4f_5/2_	∼162.68	19.22	∼164.15	09.07	∼164.30	07.60
Br	3d_5/2_	∼68.05	20.31	-	-	-	-
	3d_3/2_	∼69.32	19.31	-	-	-	-
I	3d_5/2_	-	-	∼ 630.39	14.18	-	-
	3d_3/2_	-	-	∼ 619.00	06.69	-	-
Cl	2p_3/2_	-	-	-	-	∼ 199.51	30.74
	2p_1/2_	-	-	-	-	∼ 200.60	24.22

### 3.4 UV–visible and PL Spectroscopy Analysis

The optical characteristics and light-harvesting potential of Cs_3_Bi_2_ *X*_9_ perovskite were examined using UV–visible absorption spectroscopy. Figure 7a illustrates the room temperature absorption spectra of the Cs_3_Bi_2_ *X*_9_ perovskites. The absorption peak exhibits a long tail extending up to 700 nm, indicating that sub-band gap transitions may arise from surface defects in the synthesized bismuth-based halide perovskite material. The absorption spectra of the halide perovskite material indicate a slight variation in peak position and shape.

**Figure 7:**
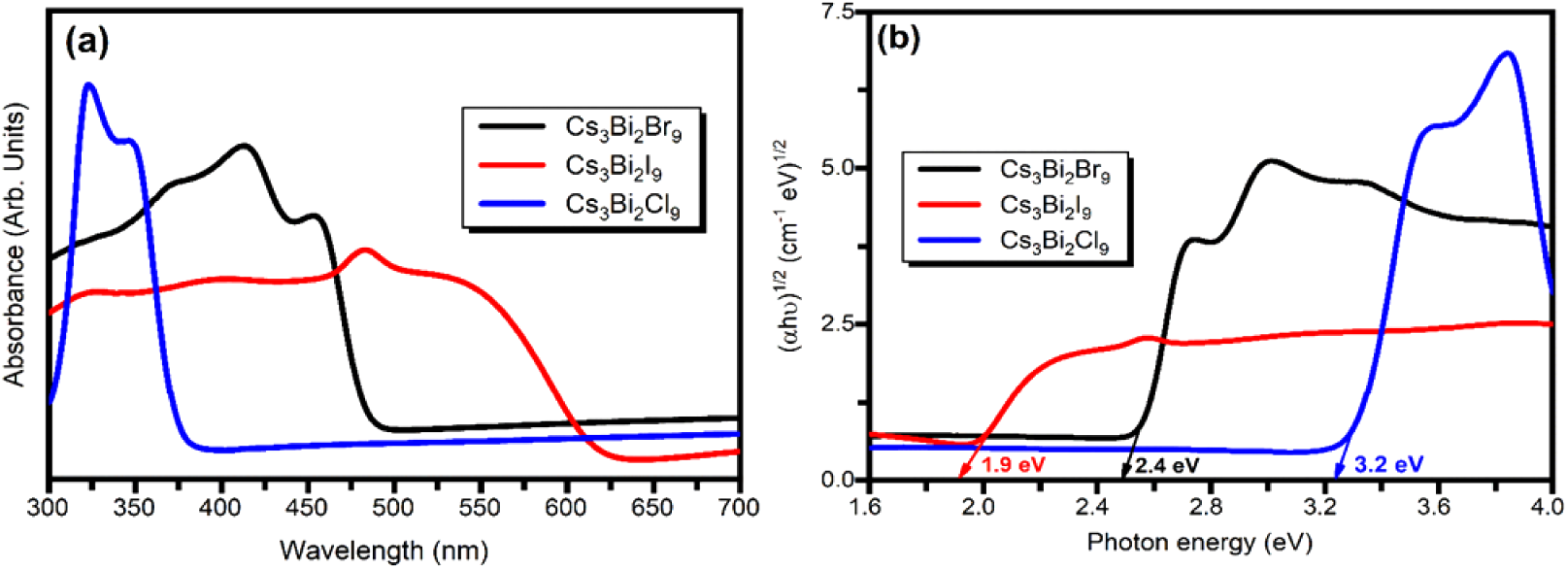
(a) UV–visible absorption spectra of Cs_3_Bi_2_ *X*_9_ (*X* = Br, I, Cl) perovskite materials and (b) Tauc’s plot for optical band gap estimation.

The optical band gap of Cs_3_Bi_2_ *X*_9_ (*X* = Br, I, Cl) perovskite was estimated using UV–vis absorption spectroscopy. The interaction between light absorption and the energy of incoming photons can be represented by the following equation,^43^

4 $${\left(\alpha h\vartheta \right)}^{1/n}=C\left(h\vartheta -E_{g}\right)$$

In which *h* is Plank’s constant, α is the absorption coefficient, ϑ is the frequency of light, “*C*” is the proportionality constant, *E*_g_ is the band gap, and *n* is the integer, 1/2 or 2, depending on the material and direct or indirect band gap. Figure 7b represents the Tauc’s plot for Cs_3_Bi_2_ *X*_9_ (*X* = Br, I, Cl) optical band gap estimation. The calculated values of the band gap for Cs_3_Bi_2_ *X*_9_ (*X* = Br, I, Cl) perovskite using the Tauc plot are listed in Table 3.

**Table 3:** Calculated Band Gap, Urbach Energy Using UV-Visible Absorption Spectra

Sample	λ_Max_ (nm)	Optical band gap (eV)	Urbach energy (eV)
**Cs**_**3**_ **Bi**_**2**_ **Br**_**9**_	485	2.4	0.120
**Cs**_**3**_ **Bi**_**2**_ **I**_**9**_	680	1.8	0.099
**Cs**_**3**_ **Bi**_**2**_ **Cl**_**9**_	380	3.2	0.105

We examine the optical characteristics and evaluate the Urbach energy (*E*_u_) of Cs_3_Bi_2_ *X*_9_ perovskite materials. Structural imperfections, impurity states, and electron-photon coupling influence absorption behavior. The Urbach energy is subsequently estimated using the following equation,^44^

5 $$\alpha =\alpha_{0}\mathrm{Exp}\left(\frac{h\vartheta }{E_{u}}\right)$$

Where α is the absorption coefficient and hϑ is the photon energy. Table 3 shows the calculated band gap and Urbach energy using UV–visible absorption spectra.

Figure 8 shows the room temperature PL spectra of Cs_3_Bi_2_ *X*_9_ (*X* = Br, I, Cl) perovskite material prepared using the antisolvent recrystallization method. The PL emission spectra of Cs_3_Bi_2_ *X*_9_ perovskites show distinct peak positions at 507.0 nm for Cs_3_Bi_2_Br_9_, 662.0 nm for Cs_3_Bi_2_I_9_, and 382.4 nm for Cs_3_Bi_2_Cl_9_. These peaks correspond to their respective band gap emissions. The PL behavior of these perovskites is strongly affected by their structural characteristics, band gap values, and the presence of defect states. All three perovskites exhibit broadband PL emission, which is mainly attributed to self-trapped excitons (STEs) rather than simple free exciton recombination. Self-trapped excitons are formed when excitons interact strongly with lattice vibrations, causing them to become localized and produce broad emission bands. In the case of Cs_3_Bi_2_I_9_, the PL emission is observed in the near-infrared region ∼661 nm. This red shift, compared to Br- and Cl-based perovskites, is due to the weaker electronegativity of iodine, which results in a smaller band gap. Moreover, Cs_3_Bi_2_I_9_ shows a broader PL emission. This broadening arises from an increased contribution of defect-assisted recombination. At the same time, Cs_3_Bi_2_Cl_9_ and Cs_3_Bi_2_Br_9_ show PL peaks at shorter wavelengths (382.4 and 507.0 nm), consistent with their larger band gaps. Their emission bands are relatively narrower, indicating fewer defect states compared to the iodide counterpart.

**Figure 8:**
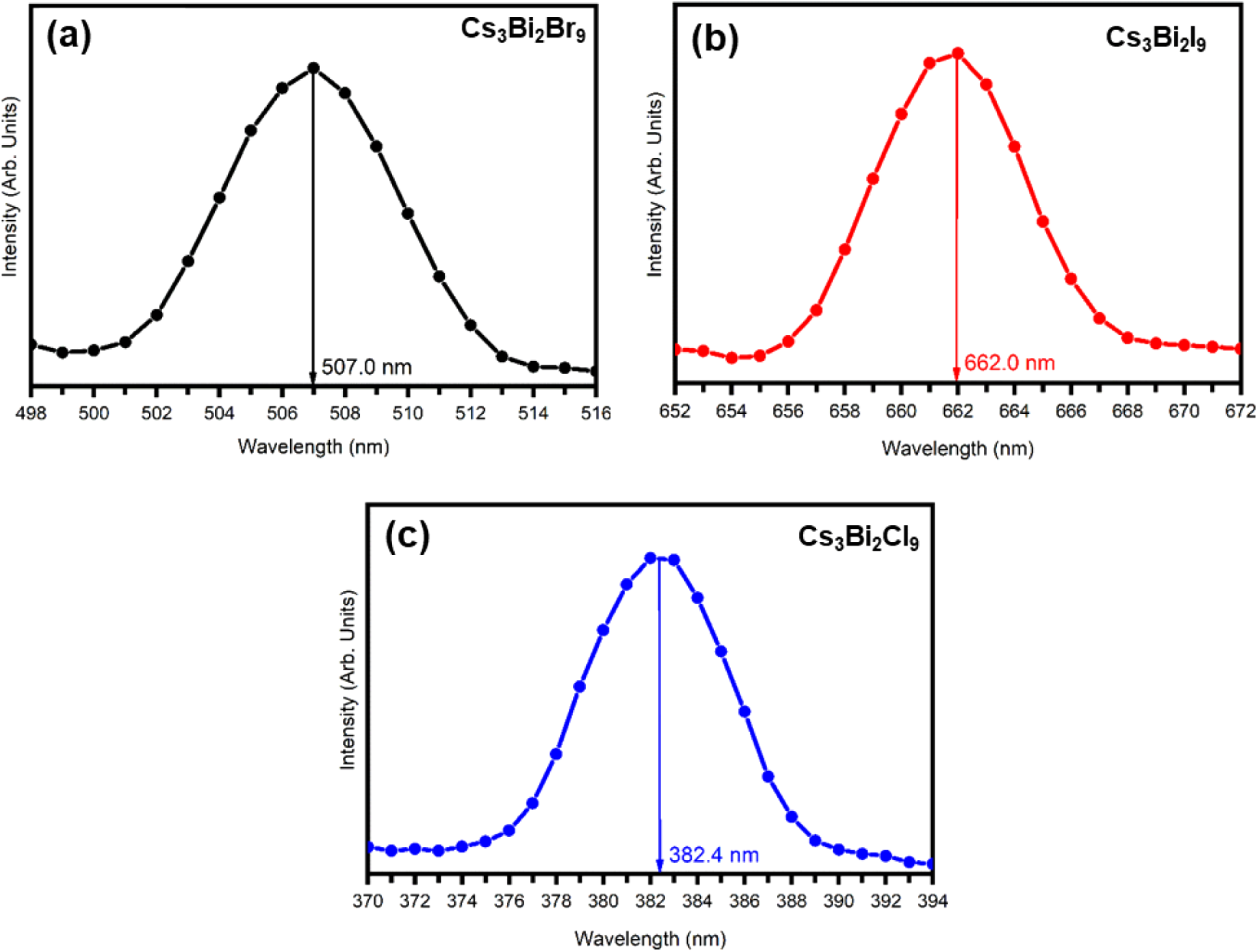
PL spectra of (a) Cs_3_Bi_2_Br_9_, (b) Cs_3_Bi_2_I_9_, and (c) Cs_3_Bi_2_Cl_9_ perovskite nanocrystals.

### 3.5 FE-SEM Analysis

The surface structure of the Cs_3_Bi_2_ *X*_9_ (*X* = Br, Cl, I) perovskites was examined using FE-SEM. The FE-SEM images of the Cs_3_Bi_2_ *X*_9_ (*X* = Br, Cl, I) perovskites are shown in Figure 9a1–c1. FE-SEM analysis showed that the particles exhibited irregular morphologies with noticeable voids across all samples. Additionally, the surfaces were predominantly covered with uneven, spherical-like granules. The surface morphology displays dense packing with evident porosity and particle sizes ranging from submicron to a few micrometers. The observed morphology supports forming a polycrystalline perovskite structure with a high surface area, which is beneficial for optoelectronic and photodetection applications. The FESEM image of the Cs_3_Bi_2_Br_9_ perovskite [Figure 9a1] demonstrates the development of several nanometer-scale facets, containing smooth, hexagonal, and irregularly round units. The FESEM image of Cs_3_Bi_2_I_9_ perovskite, shown in Figure 9b1, exhibited regularly arranged nanometer-sized spherical granules with improved density compared to Cs_3_Bi_2_Br_9_ perovskite. The agglomeration of spherical granules was observed for the Cs_3_Bi_2_Cl_9_ perovskite, as shown in Figure 9c1, resulting in a dense and compact morphology. The improved morphology may be due to higher crystallinity in Cs_3_Bi_2_Cl_9_ perovskite, which is beneficial for efficient charge transport in the photodetector devices.

**Figure 9:**
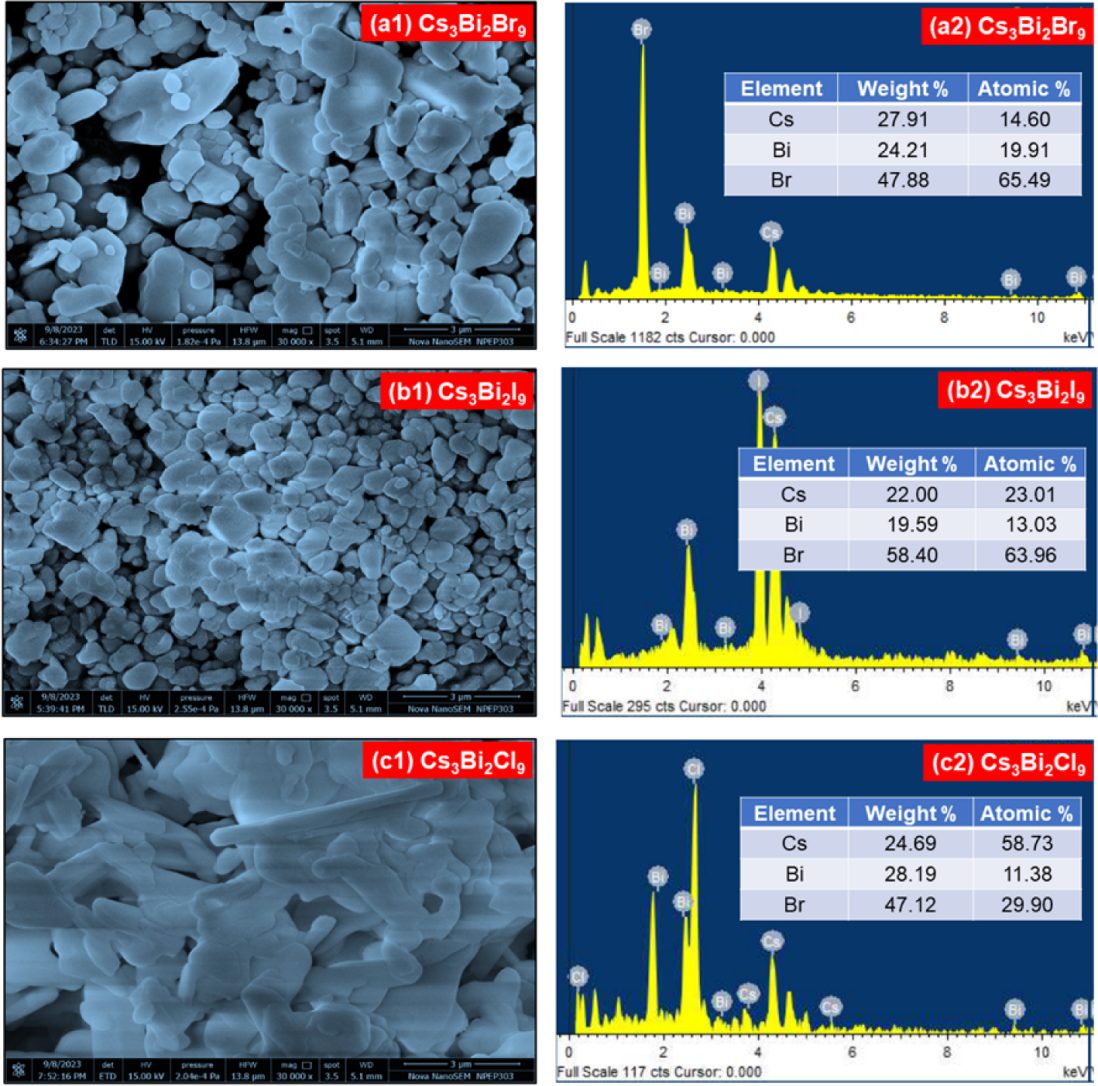
FE-SEM images of **(a1)** Cs_3_Bi_2_Br_9_, **(b1)** Cs_3_Bi_2_I_9_, and **(c1)** Cs_3_Bi_2_Cl_9_ perovskites. EDS spectra of **(a2)** Cs_3_Bi_2_Br_9_, **(b2)** Cs_3_Bi_2_I_9_, and **(c2)** Cs_3_Bi_2_Cl_9_ perovskites.

The chemical composition of the prepared films was analyzed using compositional line profile EDS. Figure 9a2–c2 displays the EDS spectra of Cs_3_Bi_2_Br_9_, Cs_3_Bi_2_I_9_, and Cs_3_Bi_2_Cl_9_ perovskite films. The inset tables in each spectrum show the elemental composition of Cs, Bi, I, Cl, and Br in the respective films. Consequently, the elemental ratios are nearly stoichiometric.

### 3.6 Photoresponse Properties

The architecture of the Cs_3_Bi_2_ *X*_9_ perovskite-based photodetector used in the present study comprises the following layers: FTO/c-TiO_2_/Cs_3_Bi_2_ *X*_9_ perovskite/Carbon Black, as shown in Figure 10 a. The active area of the perovskite film in each fabricated photodetector was ∼1 cm^2^. The Class ABA Solar Simulator (ORIEL Sol 2A 94022A) was used for white light illumination with a 24 mW/cm^2^ power density. The electrical measurement of Cs_3_Bi_2_ *X*_9_ perovskite-based photodetector was carried out using a Keithley 2450 source meter. Figure 10b illustrates the current density–voltage (*J*-*V*) characteristics for the Cs_3_Bi_2_ *X*_9_ perovskite-based photodetector at an applied voltage ranging from −1.5 to 1.5 V. The linear behavior of all *J*-*V* curves verifies the formation of Ohmic contacts. Among the samples, Cs_3_Bi_2_Cl_9_ exhibited the highest photocurrent density, which can be attributed to the higher electronegativity of chlorine compared to bromine and iodine. This enhancement can lead to improved energy level alignment between the c-TiO_2_ layer and the halide perovskite, facilitating more efficient electron injection into the c-TiO_2_ and increasing photocurrent. Additionally, chlorine’s higher ionization energy may enhance device stability and ensure consistent performance. All halide-based photodetectors respond rapidly to light and maintain a relatively constant and stable photocurrent over multiple cycles. Furthermore, the highest photocurrent density observed for Cs_3_Bi_2_Cl_9_-based photodetector can also be attributed to the higher crystallinity and low defect density compared to Cs_3_Bi_2_Br_9_- and Cs_3_Bi_2_I_9_-based photodetectors, as observed using XRD and PL spectroscopy analysis.

**Figure 10:**
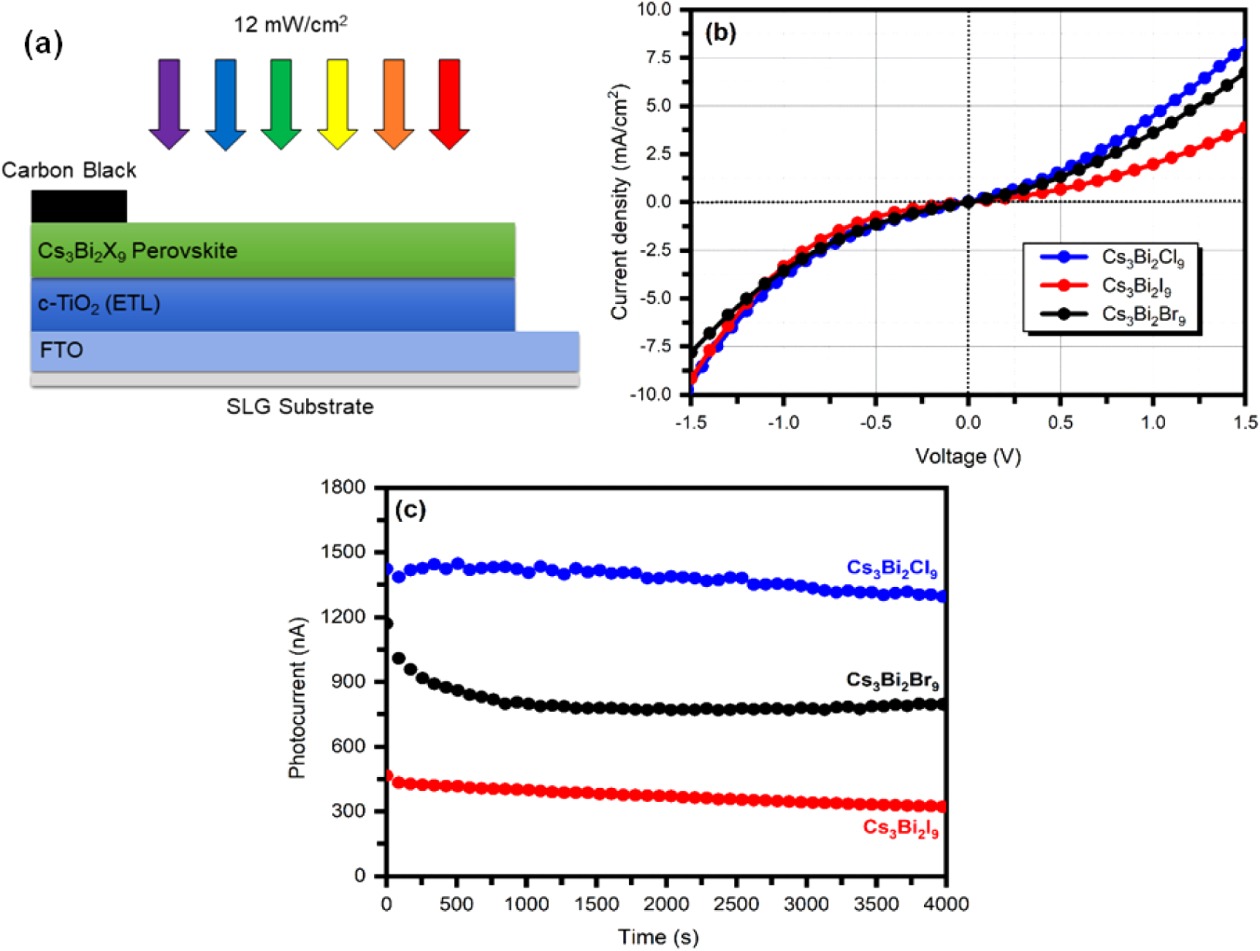
(a) Schematic of the Cs_3_Bi_2_ *X*_9_ perovskite-based photodetector used in this study, (b) *J*-*V* characteristics, and (c) stability test for the Cs_3_Bi_2_ *X*_9_ perovskite-based photodetectors.

To evaluate the stability of the photodetectors under constant bias, we performed photocurrent versus time measurements at an applied voltage of 0.5 V for Cs_3_Bi_2_Cl_9_, Cs_3_Bi_2_Br_9_, and Cs_3_Bi_2_I_9_-based photodetectors, as shown in Figure 10c. All devices displayed stable photocurrent responses over a period of 4000 s. However, the magnitude and stability vary depending on the halide composition. The Cs_3_Bi_2_Cl_9_-based photodetector device showed the highest and most stable photocurrent (>13 μA), with almost no degradation over time. The Cs_3_Bi_2_Br_9_-based photodetector device initially exhibited a photocurrent of ∼12 μA, which gradually decreased and stabilized at ∼8 μA. In contrast, the Cs_3_Bi_2_I_9_-based photodetector demonstrated the lowest stability, with its photocurrent continuously declining from ∼4.5 μA to ∼3 μA during the measurement period. These observations are consistent with the *J*-*V* characteristics shown in Figure 10b, where the Cs_3_Bi_2_Cl_9_-based photodetector exhibited the strongest photoresponse, the Cs_3_Bi_2_Br_9_-based photodetector showed moderate performance, and the Cs_3_Bi_2_I_9_-based photodetector produced the weakest response. This agreement between the two sets of measurements confirms that the halide component plays a crucial role in determining both the immediate photoresponse and the long-term operational stability of the perovskite photodetectors. The superior performance of the Cs_3_Bi_2_Cl_9_-based photodetector can be attributed to its better crystallinity and enhanced charge transport properties, which support more efficient and stable photocurrent generation. The XRD analysis further supports this.

The time-resolved photoresponse of the first five cycles of Cs_3_Bi_2_ *X*_9_-based photodetectors at 0.5 bias voltage is presented in Figure 11a1,b1 and c1. Among them, the Cs_3_Bi_2_Cl_9_-based photodetector demonstrates the highest photocurrent response. Although the chlorine-based device shows some fluctuation in current stability, it significantly enhances the overall photocurrent compared to the bromine and iodine-based photodetectors. The observed increase in photocurrent may be due to improved film crystallinity, fewer grain boundaries, and reduced defect density. The transient performance and recovery data for one cycle of the Cs_3_Bi_2_ *X*_9_-based photodetectors at 0.5 V are shown in Figure 11a2,b2 and c2. The response time (τ_Rise_) refers to the time it takes for the photodetector’s photocurrent to increase from its initial dark current level to 90% of its maximum value. Conversely, the decay time (τ_Decay_) is required for the photocurrent to drop from its peak value to 10% during the falloff phase.^45^

**Figure 11:**
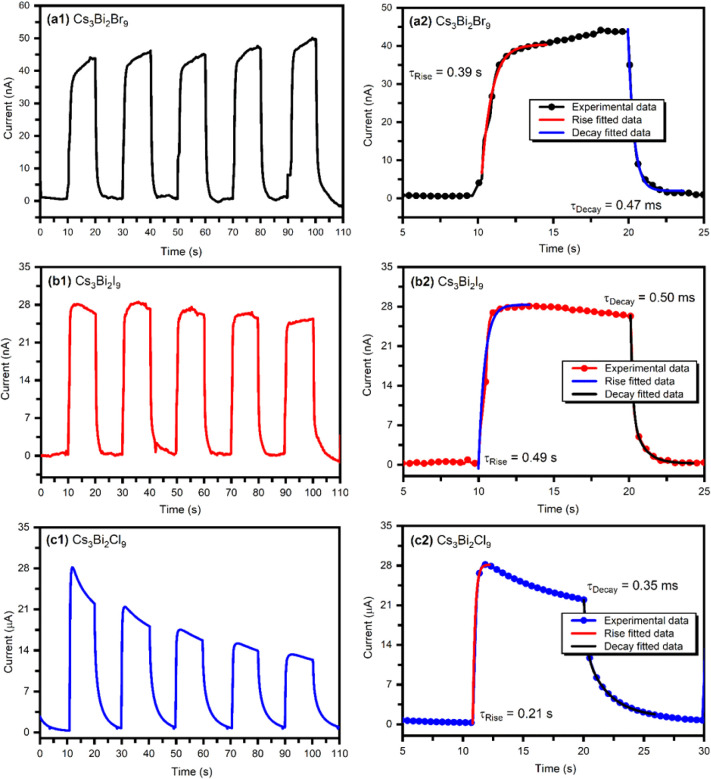
Time-resolved photoresponse of the first five cycles of **(a1)** Cs_3_Bi_2_Br_9_, **(b1)** Cs_3_Bi_2_I_9_, and **(c1)** Cs_3_Bi_2_Cl_9_ based photodetectors. The dynamic response and recovery data for one cycle of the **(a2)** Cs_3_Bi_2_Br_9_, **(b2)** Cs_3_Bi_2_I_9_, and **(c2)** Cs_3_Bi_2_Cl_9_ based photodetectors at 0.5 bias voltage.

The rise and decay of the photocurrent exhibit exponential characteristics.

6 $$I=I_{0}e^{-t/\tau_{\mathrm{Rise}}}$$

7 $$I=I_{0}e^{-t/\tau_{\mathrm{Decay}}}$$

Where *I*_0_ is the photocurrent, τ_Rise_ and τ_Decay_ are the rise and decay times, respectively, and *t* is the time constant. The estimated values of response time and decay time of Cs_3_Bi_2_ *X*_9_ photodetectors are listed in Table 4. The Cs_3_Bi_2_Cl_9_ perovskite-based photodetector exhibited the fastest response time of 0.21 s and a decay time of 0.35 ms. The Cs_3_Bi_2_Cl_9_ perovskite shows faster rise and decay times compared to Cs_3_Bi_2_Br_9_ and Cs_3_Bi_2_I_9_. We think that this improvement mainly comes from its high crystallinity and low density of trap states. When a material has good crystallinity, the crystal lattice is well-ordered, and the number of structural defects is very low. At the same time, a reduced trap density means fewer defect sites can capture charge carriers. As a result, when light is switched ON, the photogenerated charge carriers in Cs_3_Bi_2_Cl_9_ move quickly through the crystal lattice without being delayed by traps. This results in a rapid increase in photocurrent. Similarly, when the light is switched OFF, the carriers recombine instead of remaining trapped and slowly released over time. It ensures a fast decay in photocurrent. The inference is well supported by XRD analysis, which confirms the superior crystallinity of Cs_3_Bi_2_Cl_9_, and by PL studies, which indicate fewer nonradiative recombination centers or defects.

**Table 4:** Calculated Values of Rise Time (τ_Rise_), Decay Time (τ_Decay_), Photoresponsivity (*R*_λ_), Photosensitivity (ξ), Photodetectivity (D*), and Internal Quantum Efficiency (IQE) of Cs_3_Bi_2_ *X*_9_ Perovskite-Based Photodetectors

Perovskite	*R*_λ_ (mA/W)	ξ	D* (10^8^) Jones	τ_Rise_ (s)	τ_Decay_ (ms)	IQE (%)
**Cs**_**3**_ **Bi**_**2**_ **Br**_**9**_	1.84 × 10^–3^	413	3.14	0.39	0.47	0.000393
**Cs**_**3**_ **Bi**_**2**_ **I**_**9**_	1.18 × 10^–3^	176	1.64	0.49	0.50	0.000252
**Cs**_**3**_ **Bi**_**2**_ **Cl**_**9**_	1.14	58	29.6	0.21	0.35	0.245

The performance of Cs_3_Bi_2_ *X*_9_-based photodetectors can be further explored by calculating their photoresponsivity, photosensitivity, internal quantum efficiency, and detectivity. The photoresponsivity parameter indicates the amount of photocurrent produced by a photodetector per unit of incident light power. It is calculated using the following formula,^46^

8 $$R_{\lambda }=\frac{\Delta I}{P_{\lambda }\times A}$$

Where *A* is the active area of the photodetector film (1 cm^2^), Δ*I* = *I*_Photo_ – *I*_Dark_ is the difference between photocurrent and dark current, and P_λ_ is the power density of incident light (24 mW/cm^2^). The photoresponsivity increases from 1.18 × 10^–3^ for Cs_3_Bi_2_I_9_ perovskite to 1.14 mA/W for Cs_3_Bi_2_Cl_9_. These values are listed in Table 4.

Photodetectivity (D*) is a key parameter that indicates the performance of a photodetector. It is calculated using the formula,^47^

9 $$D^{*}=\frac{R_{\lambda }}{{\left(2\times e\times J_{\mathrm{Dark}}\right)}^{1/2}}$$

Where *J*_Dark_ is the dark current density and *e* is the electron charge. The calculated values of photodetectivity for perovskite-based photodetectors are listed in Table 4. The highest photodetectivity is observed for Cs_3_Bi_2_Cl_9_ perovskite-based photodetector (29.6 × 10^8^ Jones), followed by Cs_3_Bi_2_Br_9_ and Cs_3_Bi_2_I_9_ perovskite-based photodetectors.

The photosensitivity (ξ) of a photodetector is the relative change in current (Δ*I*) compared to the dark current (Idark) and expressed as,^48^

10 $$\left(\xi \right)=\frac{I_{\mathrm{Photo}-}I_{\mathrm{Dark}}}{I_{\mathrm{Dark}}}$$

The highest photosensitivity is observed for Cs_3_Bi_2_Br_9_ perovskite-based photodetector (413), followed by Cs_3_Bi_2_Cl_9_ (176) and Cs_3_Bi_2_Cl_9_ (58) perovskite-based photodetectors. Photosensitivity mainly depends on photocurrent and dark current. The highest photosensitivity observed for Cs_3_Bi_2_Br_9_ perovskite-based photodetector can be due to the higher rate of increase in its photocurrent than the dark current. Another possible reason for the highest photosensitivity observed for Cs_3_Bi_2_Br_9_ perovskite-based photodetector may be due to the high degree of crystallinity and low defect density in the Cs_3_Bi_2_Br_9_ film. From the XRD analysis, the Cs_3_Bi_2_Br_9_ film shows excellent crystallinity, with an average crystallite size of ∼50 nm and a low dislocation density (0.40 × 10^–4^ nm^–2^). These results indicate that the film contains fewer lattice imperfections. In addition, the measured microstrain (2.55 × 10^–3^) is moderate, suggesting that the internal distortion within the lattice is relatively small compared to Cs_3_Bi_2_I_9_ and Cs_3_Bi_2_Cl_9_. Such structural quality is favorable for efficient charge transport in the device. Furthermore, the Cs_3_Bi_2_Br_9_ film has a higher Urbach energy (0.120 eV) compared to Cs_3_Bi_2_I_9_ (0.099 eV), but it is close to that of Cs_3_Bi_2_Cl_9_ (0.105 eV). The higher Urbach energy suggests that the Cs_3_Bi_2_Br_9_ film has a high degree of optical disorder. However, the high degree of optical disorder is compensated for by the high crystallinity and relatively low defect density of the Cs_3_Bi_2_Br_9_ film. Thus, the high photosensitivity of Cs_3_Bi_2_Br_9_ comes from a balanced combination of good crystallinity, fewer lattice defects, and a moderate level of optical disorder. These material qualities enable the photodetector to separate charge carriers more effectively, reduce the losses through nonradiative recombination, and enhance efficient generation and transport of photocarriers under light illumination.

Internal quantum efficiency (IQE) represents the effectiveness with which a photodetector converts incident photons into electrical charge carriers under an applied bias. It is the ratio of generated charge carriers (electrons or holes) to the number of incoming photons. It is expressed as,^49^

11 $$\mathrm{IQE}\;\left(\%\right)=\frac{h\times c\times R_{\lambda }}{e\times \lambda }\times 100\%$$

Where *h* is Planck’s constant, *R*_λ_ is the photoresponsivity, *c* is the velocity of light, *e* is the charge on the electron, and λ is the wavelength of the used light.

Moreover, to illustrate the importance of this study, we have compared it with earlier research documented in the literature by examining active perovskite materials, synthesis methods, and the rise and decay times for photodetector applications. Table 5 presents several previously reported Pb-free, Cs-based perovskite photodetectors prepared using various techniques and their corresponding rise and decay times. As shown in Table 5, the antisolvent crystallized Cs_3_Bi_2_Cl_9_ perovskite-based photodetector exhibited enhanced photodetection properties compared to previously reported Cs-based perovskite photodetectors that employed different methods. This work, therefore, presents a straightforward and effective approach for designing and fabricating Pb-free, Cs-based perovskite photodetectors with exceptional photodetection response times, positioning it as a promising candidate for future photodetector applications.

**Table 5:** Photodetection Response Time of Some Previously Reported Pb-Free, Cs-Based Perovskite Photodetectors Prepared Using Different Methods

Perovskite material	Synthesis method	Rise time	Decay time	Reference
**Cs**_**3**_ **Cu**_**2**_ **I**_**5**_	Solvent Engineering method	1.61 s	0.32 s	^50^
**Cs**_**2**_ **AgBiBr**_**6**_	Pulsed Laser Deposition	0.11 ms	0.16 ms	^51^
**FAPbBr**_**3**_	Vapor Phase Growth	25 ms	-	^52^
**Cs**_**3**_ **Bi**_**2**_ **I**_**9**_	Solution Process method	90 ms	140 ms	^53^
**Cs**_**3**_ **CeBr**_**6**_	Solution-based Protocol	93 ms	345 ms	^54^
**Cs**_**3**_ **MnBr**_**5**_	Ultrasonication	20.5 ms	50.3 ms	^55^
**Cs**_**3**_ **Sb**_**2**_ **Cl**_**3**_ **Br**_**6**_	Solution Process	0.92 s	0.70 s	^56^
**Cs**_**2**_ **NaBiCl**_**6**_	Coprecipitation	16.42 ms	16.41 ms	^57^
**Cs**_**3**_ **Bi**_**2**_ **I**_**9**_	Solution Process	88.66 ms	109.3 ms	^58^
**Cs**_**3**_ **Bi**_**2**_ **I**_**6**_ **Br**_**3**_	Spin Coating	40.7 ms	21.1 ms	^59^
**Cs**_**3**_ **Cu**_**2**_ **I**_**5**_	Antisolvent Crystallization	26.2 ms	49.9 ms	^60^
**Cs**_**3**_ **Sb**_**2**_ **Br**_**9**_	Two-step CVD method	108 ms	56.2 ms	^61^
**Cs**_**3**_ **Bi**_**2**_ **Cl**_**9**_	**Antisolvent Crystallization**	**0.21 s**	**0.35 ms**	**Present work**

## 4 Conclusions

In this study, we successfully synthesized lead-free inorganic perovskite nanocrystals of Cs_3_Bi_2_ *X*_9_ (*X* = Br, I, Cl) using a simple, room-temperature antisolvent recrystallization method on soda-lime glass substrates. Comprehensive structural, morphological, and optical characterizations confirmed the formation of high-quality perovskite films with tunable properties based on halide composition. Cs_3_Bi_2_Cl_9_ exhibited the largest crystallite size, superior crystallinity, and favorable surface morphology among the three variants. The optical band edge of the Cs_3_Bi_2_ *X*_9_ materials varied between 1.9 and 3.2 eV, depending on the halide ion, offering spectral tunability for various optoelectronic applications. Photodetector devices fabricated using these films demonstrated that Cs_3_Bi_2_Cl_9_ delivered the best performance, with high detectivity (2.96 × 10^8^ Jones), photoresponsivity (1.14 mA/W), rapid response time, and internal quantum efficiency (2.45 × 10^–1^ %). These results highlight the material’s strong potential as a stable, environmentally friendly alternative to lead-based perovskites. Cs_3_Bi_2_Cl_9_ is a promising candidate for next-generation, cost-effective, and eco-friendly photodetectors. This study initiates future research and development of lead-free perovskite materials in advanced optoelectronic devices.

## Data Availability

The data sets used and analyzed during the current study are available from the corresponding author upon reasonable request.
